# Presentation of Antibacterial and Therapeutic Anti-inflammatory Potentials to Hydroxyapatite *via* Biomimetic With *Azadirachta indica*: An *in vitro* Anti-inflammatory Assessment in Contradiction of LPS-Induced Stress in RAW 264.7 Cells

**DOI:** 10.3389/fmicb.2019.01757

**Published:** 2019-08-07

**Authors:** Anusuya Nagaraj, Suja Samiappan

**Affiliations:** Department of Biochemistry, Bharathiar University, Coimbatore, India

**Keywords:** biomimetic, hydroxyapatite, *Azadirachta indica*, antioxidant, antibacterial, anti-inflammation

## Abstract

In the present study, for the first time, biomimetization of hydroxyapatite (HA) with *Azadirachta indica* (AI) was proposed and established its antioxidant, antibacterial, and anti-inflammatory potential on lipopolysaccharide (LPS). The ethanolic extract of AI was found rich with phenolics and flavonoids, and determined their concentration as 8.98 ± 1.41 mg gallic acid equivalents/g and 5.46 ± 0.84 mg catechin equivalents/g, respectively. The HA was prepared by sol-gel method from calcium nitrate tetrahydrate and orthophosphoric acid, and successfully biomimetization was performed with ethanolic extract of AI. The FTIR analysis settled that as-synthesized HA-AI composite was comprised of both HA and AI. The XRD pattern and Zeta potential revealed that the HA-AI composite was crystalline and negative in charge (−24.0 mV). The average-size distribution, shape, and size of the HA-AI composite was determined as 238.90 d.nm, spherical, and 117.90 nm from size distribution, SEM, and HR-TEM analysis, respectively. The SEM-EDX concluded that the HA-AI composite was comprised of elements of HA as well as AI. The HA-AI composite presented potential antioxidant activity and its EC50 values (dose required to inhibit about half of the radicals) for ABTS and DPPH assays were determined as 115.72 ± 2.33 and 128.51 ± 1.04 μg/ml, respectively. The HA-AI composite showed potent antibacterial activity, and minimum inhibitory concentration (MIC) and minimum bactericidal concentration (MBC) towards *S. aureus* (ATCC 700699) and *E. coli* (ATCC 10536) were correspondingly determined as 266.7 ± 28.87 and 600.0 ± 50.0 μg/ml, and 400.0 ± 86.6 and 816.7 ± 76.38 μg/ml. Most importantly, HA-AI composite presented the potential anti-inflammatory response toward lipopolysaccharide (LPS) in RAW 264.7 cells. The dose of 250 μg/ml of HA-AI composite has shown optimum protection against LPS-induced stress (1 μg/ml) by scavenging oxidants and regulating mitochondrial membrane potential (MMP), inflammatory and apoptotic factors. Thus, this study concluded that the impartation of potential biofunctional features to HA from plant sources through biomimetic approach is much beneficial and could find potential application in dentistry and orthopedic.

## Introduction

The earth is an affluent source of biominerals, such as calcium carbonates, calcium phosphates, iron hydroxides, and iron oxyhydroxides of unicellular and multicellular organisms that occur in the form of shell, ivory, teeth, magnetic crystals, etc. (Driessens and Verbeeck, 1990; Dhami et al., 2013). The biominerals possess excellent strength, fracture toughness, highly smooth finish of surface, are non-toxic and eco-friendly, and have the potential to be used as implanting agents in the dental and orthopedic fields (Cao et al., 2010). Particularly, the group of calcium apatite known as hydroxyapatite (HA) is the principal biomineral constituent and takes over 70% of weight in the bone and tooth enamel and exists in trace amount in the pineal gland and corpora arenacea (Shetty and Kundabala, 2013). HA owns great mechanical strength, biocompatibility, low resorbability, osteoconductivity, etc. Thus, HA is extensively used in orthopedic and dentistry as metallic implant coating and cavity filling material and also, as the key constituent in toothpaste and mouthwashes to remineralize artificial carious lesions (Pepla et al., 2014).

Regrettably, HA is highly biocompatible and apposite for prevalence bacterial biofilm and endotoxins (Zablotsky et al., 1992). Endotoxins, also known as lipoglycans and lipopolysaccharides (LPS), consist of lipid and polysaccharide O-antigen, and are exclusively present in the outer membrane of Gram-negative bacteria (Raetz and Whitfield, 2002). LPS induces the bulk release of inflammatory cytokines, which may result in septic shock, systemic inflammatory response syndrome, severe tissue damage, and multiple organ dysfunction (Lamping et al., 1998). With the advent of nanotechnology, the proficiency of HA as an implant in orthopedic and dentistry has been greatly boosted by doping it with chemical elements such as titanium, cobalt, magnesium, zinc, silver, and gold (Lin et al., 2007; Mo et al., 2008; Nirmala et al., 2011; Kramer et al., 2014; Gayathri et al., 2018; Sathiskumar et al., 2018). However, these chemical elements detrimentally interact with blood cells, including erythrocytes, leukocytes, platelets, and macrophages due to the generation of oxidative stress and inflammatory response (Asharani et al., 2010; Chen et al., 2015). Typically, these chemical elements form fibrous local pseudocapsules interacting with fibroblasts and deposit in the local tissues and organs. Also, these chemical substances translocate and disseminate into the key organs such as liver, spleen, lung, and kidney *via* blood circulation and constitute a health risk (Kim et al., 2010; Khan et al., 2012; Wang et al., 2016). Insight of the aforementioned demerits, biomimetic of HA composite with potent antibacterial and anti-inflammatory bioresources could be highly preferred and recommended over chemical substances for orthopedic implants. In the current scenario, plant sources are highly preferred due to their non-toxicity and eco-friendliness (Kalaiselvi et al., 2018; Phatai et al., 2019). Therefore, the biomimetic of HA with plant sources could be highly appropriate and satisfactory.

To the best of our knowledge, the therapeutic potential of the biomimetic HA composite with plant sources on inflammatory response has not been documented so far, so this would be the first investigation. In the present study, HA was prepared by sol-gel method from calcium nitrate tetrahydrate and orthophosphoric acid. Furthermore, biomimetic of HA with ethanolic extract of *Azadirachta indica* (AI) was undertaken. AI is an ancient herbal medicinal plant that belongs to the *Meliaceae* family and is widely recommended in a variety of biological uses such as anti-allergic, antimicrobial, antimalarial, antiulcer, antitumor, insecticide, and pesticide (Biswas et al., 2002; Pankaj et al., 2011; Alzohairy, 2016).

The synthesized HA and biomimetic HA-AI composite were characterized by FTIR, XRD, Zeta potential and size distribution, SEM with EDX, and HR-TEM analysis. The *in vitro* radical scavenging potential of HA-AI composite was studied by DPPH and ABTS assays. The *in vitro* antibacterial activity of the HA-AI composite against pathogenic bacteria was determined in minimum inhibitory concentration (MIC) and minimum bactericidal concentration (MBC) by micro-well dilution technique. Furthermore, detrimental micro-morphological changes induced in pathogenic bacteria by the HA-AI composite were registered by SEM observation. The *in vitro* anti-inflammatory response of the HA-AI composite against endotoxin LPS was assessed in macrophages (RAW 264.7 cells) by determining cell viability, oxidative and mitochondrial stress, inflammatory, and caspase-3 levels.

## Materials and Methods

### Chemicals and Reagents

Ethanol (99.80%), 2,2-diphenyl-1-picrylhydrazyl (DPPH), Muller-Hinton agar (MHA), Muller-Hinton broth (MHB), 2,2′-azino-bis (3-ethylbenzothiazoline-6-sulphonic acid) (ABTS), and fetal bovine serum (FBS) were received from HiMedia, Mumbai, India. Calcium nitrate tetrahydrate, orthophosphoric acid, gallic acid, catechin, rutin, Dulbecco’s modified Eagle’s medium (DMEM), antibiotic and antimycotic solution, lipid peroxidation kit, Griess reagent, caspases kits (3/7, 8, and 9), antioxidant enzyme kits (SOD, CAT, and GSH), ELISA kits (TNF-α and IL-6), primer sequences (TNF-α, IL-6, iNOS, COX-2, and β-actin), dichloro-dihydro-fluorescein diacetate (DCFH-DA), TRI reagent, rhodamine 123, and 4′,6-diamidino-2-phenylindole (DAPI) were purchased from Sigma-Aldrich, Bengaluru, India. The live/dead dual staining kit was purchased from Thermo Fisher Scientific and iScript One-Step RT-PCR kit with SYBR green was obtained from Bio-Rad, Bengaluru, India.

### Collection, Preparation, and Phytochemical Analysis of Plant Material

AI was collected from Bharathiar University Campus, Coimbatore, India. The voucher was authenticated in Department of Botany, Bharathiar University, and safeguarded. The stem was detached and washed twice with double distilled water and dried under shade at room temperature for 2 weeks. Following, 500 g was ground to a fine powder by electrical blender, and active components were extracted by ethanol solution following cold maceration technique (Pandey et al., 2014). The attained ethanolic extract was concentrated by lyophilization (−39°C) and stored in a screw-capped amber glass vial at 4°C for further use.

#### Determination of Total Phenolics

The total phenolics of AI extract were determined by Folin-Ciocalteu assay (Kalagatur et al., 2018). In brief, different concentrations of AI extract were supplemented to 0.5 ml of 7.5% sodium carbonate solution and 0.25 ml of Folin-Ciocalteu reagent and incubated in the dark at room temperature for 30 min. Subsequently, optical density (OD) was monitored at 765 nm using the multimode plate reader (Synergy H1, BioTek, USA). Gallic acid (GA) was considered as the reference standard and the calibration curve was constructed. The total phenolics of AI extract were derived from the calibration curve of GA and the result was expressed as milligrams of GA equivalents per gram of AI extract (mg GAE/g).

#### Determination of Total Flavonoids

The determination of total flavonoids of AI extract was undertaken by aluminum chloride colorimetric method (Kalagatur et al., 2018). Briefly, different concentrations of AI extract were supplemented to 70 μl of sodium nitrite solution (5%) and allowed to rest for 5 min before being combined with 1.3 ml of distilled water, 0.5 ml of sodium hydroxide (1 M), and 0.15 ml of aluminum chloride (10%) and kept at room temperature for 5 min. Ensuing, OD was monitored at 415 nm using a multimode plate reader (Synergy H1, BioTek, USA). Catechin (CC) was considered as the reference standard and a calibration curve was constructed. The total flavonoids of AI extract were derived from the calibration curve of CC and the result was expressed as milligrams of CC equivalents per gram of AI extract (mg CCE/g).

### Preparation of Hydroxyapatite

HA was prepared by sol-gel process as described before (Sanosh et al., 2009) with minor modifications. Calcium nitrate tetrahydrate (CNT) and orthophosphoric acid (PA) were used as the precursors. A solution of PA (0.25 M) was prepared in distilled water and ammonia was added by continuous stirring until the pH adjusted to 10. The ratio of Ca/P was maintained at ~1.67 by adding CNT solution (1 M in distilled water) and the solution was vigorously stirred at 200 rpm for 1 h. The solution was subjected to aging for overnight and dried at 65°C in a hot air oven.

The obtained powder was washed repeatedly with distilled water to remove NH_4_ and NO_3_ and calcined in the electrical furnace at 800°C for 1 h. The HA powder was packed in amber glass vial and stored at room temperature for further purposes.

The following reactions occur in the formation of HA during sol-gel preparation,

(1) $$H_{3}{PO}_{4}+3{NH}_{4}OH\to {\left({NH}_{4}\right)}_{3}{PO}_{4}+3H_{2}O$$

(2) $$\begin{array}{l} 6{\left({NH}_{4}\right)}_{3}{PO}_{4}+10Ca{\left({\mathrm{NO}}_{3}\right)}_{2}\cdot 4H_{2}O\to \;{Ca}_{10}{\left({PO}_{4}\right)}_{6}{\left(OH\right)}_{2}\\ \;+20{NH}_{4}{\mathrm{NO}}_{3} \end{array}$$

### Preparation and Characterization of HA-AI Composite

The biomimetic of HA with AI, and thus the preparation of HA-AI composite, was done previously described (Bismayer et al., 2005) with slight modifications. The ethanolic extracts of AI and HA were blended (400:400 mg, w/w) in 40 ml of ethanol, incubated overnight at 120 rpm, and dried out in hot air oven at 25–30°C. The prepared HA-AI composite was stored in an amber glass vial at 4°C in a dry place and used for further studies.

The physicochemical analysis of the prepared HA and HA-AI composite was assessed by various techniques, including Fourier transform infrared spectroscopy (FTIR), X-ray diffraction (XRD), Zeta potential and size distribution, scanning electron microscope-energy dispersive X-ray (SEM-EDX), and high-resolution transmission electron microscopy (HR-TEM) analysis. The FTIR analysis was performed to confirm the successful frame-up of the HA-AI composite. The samples were pelletized with KBr and IR transmission spectra were recorded in attenuated total reflectance mode (ATR) from 4,000 to 400 cm^−1^ at a data acquisition rate of 2 cm^−1^ using FTIR 84005 (Shimadzu, Tokyo, Japan). The XRD analysis was done to understand the nature of the HA and HA-AI composite using Ultima IV diffractometer (Rigaku, USA) with Cu Kα radiation (*λ* = 1.541 Å) in 2*θ* range of 10°–80° with scan rate of 3°/min. The crystallite pattern of HA and HA-AI composite was calculated from the standard XRD pattern of the International Centre for Diffraction Data (ICDD). The surface charge and size distribution of the HA and HA-AI composite were measured using Zeta Sizer ZS 90 (Malvern Instruments, Germany) with He-Ne laser beam at 532 nm wavelength in backscattering mode. The surface microstructure and elemental composition of HA and HA-AI composite were examined using SEM-EDX (FEI, Quanta 200, Thermo Fisher Scientific, USA). The shape and size of the HA and HA-AI composite were confirmed by HR-TEM.

### Antioxidant Potential of HA-AI Composite

The radical scavenging potential of the HA-AI composite was assessed by DPPH and ABTS radical scavenging activity (Kumar et al., 2016). In case of DPPH assay, a stable DPPH radical solution was prepared by dissolving 2.4 mg of DPPH in 100 ml of methanol. For ABTS assay, an ABTS radical solution was prepared by incubating the reaction mixture of 7 mM ABTS in water and 2.45 mM potassium persulfate in water (1:1, v/v) for 12–16 h in the dark at room temperature. The ABTS radical solution was diluted with methanol to attain the optical density (OD) of 0.70 at 734 nm. Following, different concentrations of the HA-AI composite were prepared in methanol and 100 μl was added to 2.9 ml of DPPH and 3.9 ml of ABTS radical solution. The blend was shaken vigorously and incubated for 30 min in the dark at room temperature. The ODs of DPPH and ABTS blends were determined using multiplate reader (Synergy H1, BioTek, USA) at 517 and 734 nm, respectively. The radical solution alone was considered as control and rutin was used as standard antioxidant compound. The percentage of radical scavenging activity was estimated using the formula

$$\begin{array}{l} \mathrm{DPPH}\;\mathrm{or}\;\mathrm{ABTS}\;\mathrm{radical}\;\mathrm{scavenging}\;\mathrm{activity}\;\left(\%\right)\\ =\frac{A_{t}}{A_{c}}\;\times \;100 \end{array}$$

where *A*_c_ and *A*_t_ were absorbance of control and test sample, respectively.

### Microbicidal Activity of HA-AI Composite

The microbicidal activity of the HA-AI composite was determined by micro-well dilution method as per the Clinical & Laboratory Standards Institute (CLSI) (Qaiyumi, 2007). The selected pathogenic bacteria, *Staphylococcus aureus* (ATCC 700699) and *Escherichia coli* (ATCC 10536), were obtained from the American Type Culture Collection (ATCC), USA. The bacteria were grown overnight in MHB at 37°C, and OD was determined at 600 nm using a microplate reader (Synergy H1, BioTek, USA) and OD of broth culture was adjusted to 0.5 McFarland standard with sterile PBS pH 7.4. A quantity of 10 μl broth culture (0.5 McFarland standard) and different concentrations of HA-AI composite were added to the wells of the microtiter plate and the final volume was adjusted to 100 μl with MHB and incubated for 24 h at 37°C. The wells containing only bacteria without HA-AI composite were considered as control and tetracycline was used as a reference standard. Following the incubation period, OD was measured at 600 nm and the concentration of the HA-AI composite, at which an increase in OD (bacterial growth) is not registered, was stated as minimum inhibitory concentration (MIC). Subsequently, a volume of 10 μl was collected from each well of the microtiter plate and spread plated on MHA Petri plates and incubated for 24 h at 37°C. The concentration of HA-AI composite at which bacterial growth did not reoccur was specified as MBC.

Concurrently, 10 μl of control and test bacterial samples were collected from microtiter plate and heat fixed to the glass slide. The bacterial samples were fixed with the gradient solutions of glutaraldehyde (5, 10, and 15%) and sputter coated with gold-palladium as per the technique described by Kalagatur et al. (2015). The micro-morphology of bacteria was photographed using SEM (FEI, Quanta 200, Thermo Fisher Scientific, USA) at 20 kV in environmental mode.

### Therapeutic Potential of HA-AI Composite on LPS-Induced Inflammatory Stress

#### Cell Culture and Treatments

The therapeutic potential of the HA-AI composite on LPS-induced inflammatory stress was revealed in RAW 264.7 cells (macrophages) of *Mus musculus*. The RAW 264.7 cells were acquired from the National Centre for Cell Science (NCCS, Pune, India) and maintained in DMEM supplemented with 10% FBS, penicillin (100 U/ml), and streptomycin (100 μg/ml) in humidified atmospheric conditions of 5% CO_2_ and 95% air at 37°C. The cells were grown in 75 cm^2^ cell culture flasks, and media was regularly changed on alternate days, and confluent cells were utilized for the experiments. The stock solution of the HA-AI composite was prepared in dimethyl sulfoxide (DMSO) and the final concentration of DMSO in the experimental sample was not higher than 0.01%.

To test the anti-inflammatory effect of HA-AI composite on LPS-induced stress, 1.5 × 10^4^ cells were seeded in 96-well cell culture plates and allowed to settle overnight. The cells were pre-treated with different concentrations of the HA-AI composite for 12 h and exposed to 1 μg/ml of LPS for 24 h in DMEM devoid of FBS. In literature, several studies prominently produced inflammatory response in *in vitro* cell line model with 1 μg/ml of LPS (Alvarez-Suarez et al., 2017; Gasparrini et al., 2017). Therefore, in the present study, 1 μg/ml of LPS was used to stimulate the inflammatory response in RAW 264.7 cells. The cells were treated with the same concentration of DMSO in which the HA-AI composite prepared was considered as control. Following, plates were distinctly employed for cell viability, oxidative and inflammatory stress, mitochondrial membrane potential (MMP), and apoptosis analysis.

Most importantly, before undertaking a detailed assessment of oxidative and inflammatory stresses, MMP, and apoptosis. The cytotoxic effect of different concentrations of HA and HA-AI composite on cell viability for 24 h were judged by MTT assay. Following, the protective efficacy of HA and HA-AI composite on LPS (1 μg/ml) induced cell death were distinctively studied by cell viability assays (MTT and live/dead) and superlative protective demonstrative on LPS-induced cell death was chosen for detailed investigations.

#### Cell Viability Analysis

##### MTT Assay

MTT assay measures the cell viability based on the redox potential of the cell. Following the cell culture and treatments stage, media were replaced with 100 μl of MTT reagent (5 mg/ml in DPBS) and incubated for 3 h at room temperature. Next, the MTT reagent was substituted with 100 μl of DMSO and kept for 30 min to solubilize the formazan crystals. The OD was measured at 570 nm using multiplate reader (Synergy H1, BioTek, USA). The results were stated with respect to control (100%) (Venkataramana et al., 2014).

##### Live/Dead Dual Staining Assay

The percentage of live and dead cells was determined by the live/dual staining technique consisting of calcein AM and ethidium homodimer-1. The calcein AM reacts with intracellular esterases of live cells and appears green in color. Whereas, ethidium homodimer crosses through damaged cellular membrane of dead cells and binds with the nuclei to produce red color (Haugland et al., 1994). After the cell culture and treatment stage, cells were washed with DPBS and stained with 2 μM of calcein AM and 4 μM of ethidium homodimer-1 for 15 min and washed with DPBS twice. The OD was recorded using multiplate reader (Synergy H1, BioTek, USA) as per instructions from the manufacturer. The percentage of live/dead cells was computed using a technique previously described (Kalagatur et al., 2017). Furthermore, images were captured using the inverted fluorescence microscope (EVOS FLC, Thermo Fisher Scientific, USA).

#### Analysis of Oxidative and Inflammatory Stress

##### Estimation of Reactive Oxygen Species

The contents of intracellular reactive oxygen species (ROS) were determined by DCFH-DA staining (LeBel et al., 1992; Kalagatur et al., 2017). After the cell culture and treatment phase, cells were incubated with 5 μM of DCFH-DA for 15 min and washed twice with DPBS. The fluorescence intensity was recorded at an excitation of 495 nm and an emission of 550 nm using the microplate reader (Synergy H1, BioTek, USA). The standard curve for ROS released versus H_2_O_2_ constructed and used to express the amount of ROS released. The results were expressed with respect to control (100%). Moreover, microscopic images of cells were captured using phase contrast and green fluorescent protein (GFP) filters of an inverted fluorescence microscope (EVOS FLC, Thermo Fisher Scientific, USA).

##### Estimation of Nitrite

The nitrite (NO) content was determined using Griess reagent. After the cell culture and treatments phase, 100 μl of the cell culture supernatant was collected and added to an equal volume of Griess reagent according to the instructions from the manufacturer. The absorbance was monitored at 550 nm using a multiplate reader (Synergy H1, BioTek, USA). The standard curve of sodium nitrite (NaNO_2_) constructed and amount of NO released were expressed as μM of NaNO_2_ (Giustarini et al., 2008).

##### Estimation of Lipid Peroxidation

After cell culture and treatments, cells were lysed and absorbance of malondialdehyde (MDA), an indicator of lipid peroxidation, was measured at 532 nm using a multiplate reader (Synergy H1, BioTek, USA) as per instructions from the manufacturer. The standard curve for MDA was constructed and the amount of MDA released was expressed as nM MDA (Gaweł et al., 2004).

##### Estimation of Antioxidant Enzymes

After cell culture and treatments, cells were lysed using the cell lysis buffer and supernatant was collected by centrifugation at 12,000× *g* for 10 min at 4°C and used for estimation of antioxidant enzymes (SOD, CAT, and GSH) using enzyme analysis kits (Sigma-Aldrich). The methodology was performed as manufacturer’s instruction (Djordjevic et al., 2004).

##### Estimation of TNF-α and IL-6

The inflammatory markers, TNF-α and IL-6, were measured using ELISA kits. After “cell culture and treatments”, supernatant was collected and treated with reagents of TNF-α and IL-6 of ELISA kits as per manufacturer’s instructions and OD was measured at 450 nm using multiplate reader (Synergy H1, BioTek, USA) (Bienvenu et al., 1993).

##### Real-Time PCR Analysis

The inflammatory regulatory genes, TNF-α, IL-6, iNOS, and COX-2, were quantified by RT-PCR analysis. The β-actin was used as reference gene (Supplementary Table S1). Following cell culture and treatments, total RNA was extracted from cells using TRI reagent as per manufacturer’s instructions and RNA purity and quantity were determined measuring OD at 260/280 nm using NanoDrop 8,000 Spectrophotometer (Thermo Fisher Scientific, USA). The RT-PCR analysis was carried out using iScript One-Step RT-PCR kit as per manufacturer’s instructions employing Light cycler 480 (Roche, USA). Briefly, the total reaction mixture of 50 μl consisted of 1 μl of primer (450 nM) and template RNA (100 ng), 1 μl of iScript reverse transcriptase for one-step RT-PCR, 25 μl of 2X SYBR Green RT-PCR reaction mix, and 22 μl of nuclease-free water (PCR grade). The RT-PCR analysis consists of 10 min of cDNA synthesis at 50°C for one cycle, 5 min of polymerase activation at 95°C and followed by 35 cycles of PCR at 95°C for 10 s, and finally 60°C for 30 s for combined annealing and extension. The relative fold expression of test genes was measured with respect to the normalized reference gene (Tellman and Olivier, 2006).

#### Estimation of Mitochondrial Membrane Potential

The MMP was determined by rhodamine 123 staining technique. After cell culture and treatments, cells were stained with rhodamine 123 for 15 min and washed with DPBS twice, and fluorescence intensity was recorded at excitation of 511 nm and emission of 534 nm under a microplate reader (Synergy H1, BioTek, USA) (Emaus et al., 1986; Kalagatur et al., 2018). The microscopic images of cells were captured using phase contrast and GFP filters of an inverted fluorescence microscope (EVOS FLC, Thermo Fisher Scientific, USA).

#### Analysis of Apoptosis

##### DAPI Staining

The nuclear content of cells was observed by DAPI staining. After cell culture and treatments, cells were stained with 1 μM of DAPI for 15 min and washed twice with DPBS. The images were captured under DAPI filter using the inverted fluorescence microscope (EVOS FLC, Thermo Fisher Scientific, USA). At least 1,000 cells were considered for apoptotic analysis in 10 different microscopic fields and results were expressed as apoptotic bodies/100 cells (Tan et al., 2006; Kalagatur et al., 2018).

##### Caspase-3/7, 8, and 9 Analysis

After cell culture and treatments, cells were individually treated with fluorimetric reagents of caspase-3/7, 8, and 9 analysis kits as per manufacturer’s instructions and optical density was recorded at excitation of 360 nm and emission of 460 nm using a multiplate reader (Synergy H1, BioTek, USA) (Cohen, 1997; Tan et al., 2006; Kalagatur et al., 2018).

### Statistical Analysis

The phytochemical, antioxidant, antimicrobial, and anti-inflammatory analyses were performed individually in six replicates and the achieved results were stated as mean ± standard deviation. The data were statistically evaluated by one-way ANOVA following Tukey’s *post hoc* multiple comparison test. The statistical variance among the experimental groups was measured as significant at *p* < 0.05.

## Results and Discussion

### Phytochemical Analysis of AI Extract

**Figure 1:**
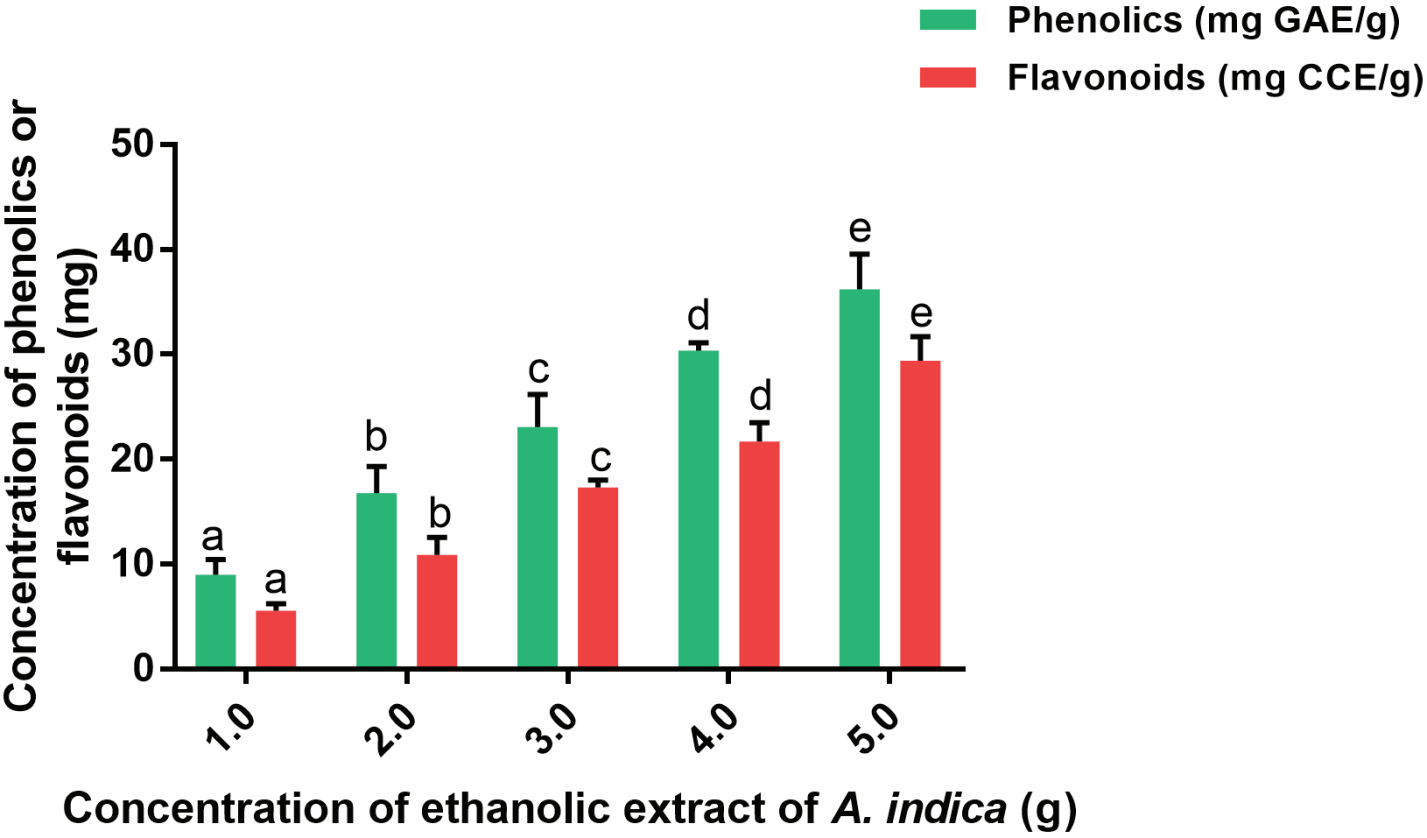
Total phenolics (mg GAE/g) and flavonoids (mg CCE/g) in ethanolic extract of *A. indica* (AI). The experiments were performed individually in six replicates and achieved results were stated as mean ± standard deviation. The data were statistically evaluated by one-way ANOVA following Tukey’s *post hoc* multiple comparison test. The statistical variance among the experimental groups was measured as significant at *p* < 0.05. The columns with different alphabets were significant within the respective study.

The successful biomimetic of nanomaterials with plant extract depends on their secondary metabolites, i.e., phenolics and flavonoids. The phenolics and flavonoids assist in reducing and stabilizing agents in biomimetic of nanomaterials (Ahmed et al., 2016). Hence, in the present study, phenolics and flavonoids were estimated. The obtained ethanolic stem extract of AI was found affluent with phenolics and flavonoids, and their concentrations were noted as 8.98 ± 1.41 mg GAE/g and 5.46 ± 0.84 mg CCE/g, respectively (Figure 1). These results were in accordance with previous documented literature. However, the concentrations of phenolics and flavonoids in our study were quite varied compared to existing literature. Al-Jadidi and Hossain (2016) determined total phenolics and flavonoids in AI stem extract to be in the range of 20.80–107.29 mg/100 g and 136.50–484.50 mg/100 g, respectively. Furthermore, Choudhary and Swarnkar (2011) determined total phenolics and flavonoids in stem bark to be 42.12 ± 1.08 mg GAE/g and 08.08 ± 0.62 mg quercetin equivalents (QE)/g, respectively. By contrast, Choudhary and Swarnkar (2011) have documented quite diverse quantities of total phenolics and flavonoids in leaves extract and determined them to be 08.83 ± 0.35 mg GAE/g and 07.82 ± 0.39 mg QE/g. In another study, Sultana et al. (2007) used different solvent extraction techniques (ethanol, methanol, and acetone) and determined the total phenolics and flavonoids in AI bark in the range of 9.30 ± 0.37–12.0 ± 0.36 mg GAE/g and 2.52 ± 0.10–3.31 ± 0.16 mg CCE/g, respectively. The quality and quantity of secondary metabolites of plant depend on genome, part of the plant (leaves, stem, flower, fruit, and root), climatic conditions, nutrients, and as well extraction technique (Sultana et al., 2007; Choudhary and Swarnkar, 2011). Hence, might be the concentration of phenolics and flavonoids were varied in our study. To conclude, the obtained ethanolic stem extract of AI was highly suitable for biomimetic of nanomaterials.

### Physicochemical Characterization of HA-AI Composite

In the present study, HA was successfully synthesized by sol-gel approach using CNT and PA as precursors. The synthesized HA was washed thoroughly with distilled water to get rid of NH_4_ and NO_3_ and effectively calcined at 800°C. Furthermore, biomimetization of HA with AI was undertaken and attained combination was dried at 25–30°C and used in further determinations.

**Figure 2:**
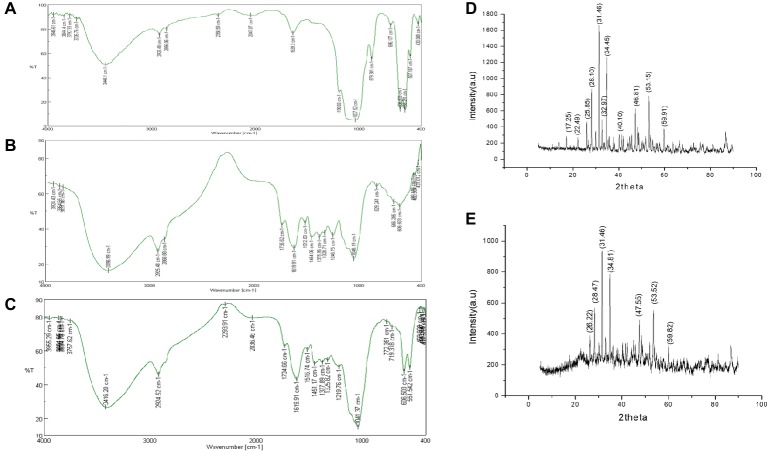
FT-IR spectrum of **(A)** hydroxyapatite (HA), **(B)** ethanolic extract of *A. indica* (AI), and **(C)** hydroxyapatite–*A. indica* (HA-AI) composite. XRD pattern of **(D)** HA and **(E)** HA-AI composite.

FTIR spectrum was recorded in the range of 4,000–400 cm^−1^ and the infrared peaks of HA, AI, and HA-AI composite were depicted in Figure 2. In HA, peaks at 558.291, 606.503, 696.177, 879.381, and 1,037.52 cm^−1^ show the characteristic presence of the phosphate group of HA. Furthermore, C=C stretching of alkene group was present at 1,639 cm^−1^ and the peak at 2,047.07 cm^−1^ corresponds to carbonyl stretch. The broad peaks which subsist between 2,856.06 and 3,448.1 cm^−1^ conform to the presence of OH (Figure 2A; Sanosh et al., 2009; Kramer et al., 2014), while AI had exhibited characteristic peaks at 1,046.19, 1,246.75, 1,328.71, 1,375.96, 1,512.88, 1,619.91, 1,735.62, 2,925.48, and 3,396.99 cm^−1^, which corresponds to amines, carboxylic acids, aromatic compounds, phenols, nitro compounds, alkenes, aldehydes, alkanes, and alcohol of phytochemical constituents of AI stem extract (Figure 2B). Whereas, the HA-AI composite presented characteristic functional groups of both HA and AI and concluded the successful formation of the HA-AI composite (Figure 2C).

The XRD pattern of the synthesized HA and HA-AI composite was given in Figures 2D,E. The majority of the diffraction peaks for HA appeared at 2θ values of 17.25, 22.49, 25.85, 28.10, 31.46, 32.97, 34.45, 40.10, 46.81, 53.15, and 59.91 were assigned to corresponding planes of 110, 111, 002, 102, 211, 300, 202, 221, 222, 004, and 420 (Figure 2D). The presence of a sharp peak at 31.46 caused by 211 plane confirmed the formation of HA with pure crystalline phase. The obtained planes were well-matched with the standard ICDD File no: 09-169 and 09-432 (Kaygili et al., 2014; Atak et al., 2017; Gayathri et al., 2018). Whereas, the HA-AI composite exhibited slight shifting in the diffraction peaks at 2*θ* values of 26.22, 28.47, 31.46, 34.81, 47.55, 53.52, and 59.82. The observed shifting could be due to the accumulation of AI within HA (Figure 2E). The XRD pattern of the HA-AI composite clinched that no noticeable change was observed in crystallinity of HA due to the accumulation of AI. However, the intensity of the diffraction peaks was reduced in HA-AI composite compared to HA due to the accumulation of phytochemical constituents of AI. In support of the present study, and earlier report (Gopi et al., 2013) concluded that the biomimetic of HA composites with plant extracts does not change the crystallinity of HA.

**Figure 3:**
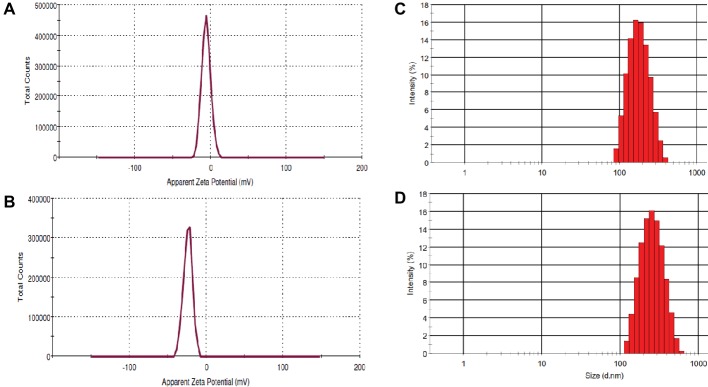
Zeta potential of **(A)** hydroxyapatite (HA) and **(B)** hydroxyapatite–*A. indica* (HA-AI) composite. DLS pattern of **(C)** HA and **(D)** HA-AI composite.

Zeta potential measurement is carried out to determine the colloidal long-term stability and surface charge of the particles and results were depicted in Figure 3. The Zeta potentials of HA and HA-AI composite in ethanol were determined as −5.87 and −24.0 mV, respectively, and concluded that the HA-AI composite was highly stable than HA (Figures 3A,B). The HA-AI composite with negative Zeta potential could be a highly appropriate implanted material intended for the maintenance of viable cells in biological systems (Chen et al., 2009). Next, dynamic light scattering (DLS) pattern revealed the average size distribution of HA and HA-AI composite were at 186.54 and 238.90 d.nm, respectively (Figures 3C,D). The polydispersity index of 0.130 and 0.227 were correspondingly noticed for HA and HA-AI composite, which was recognized as the lesser agglomeration.

**Figure 4:**
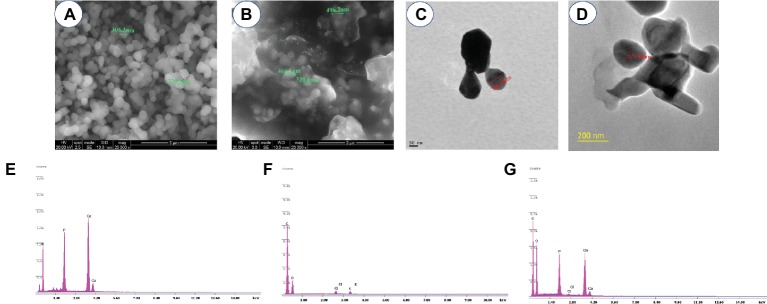
SEM images of **(A)** hydroxyapatite (HA) and **(B)** hydroxyapatite–*A. indica* (HA-AI) composite. HR-TEM image of **(C)** HA and **(D)** HA-AI composite. SEM-EDX pattern of **(E)** HA, **(F)** AI, and **(G)** HA-AI composite.

Figure 4 shows the morphology and size of HA and HA-AI composite. The SEM showed that the size of HA and HA-AI composite was in the range of 306–320 nm and 320–496 nm, respectively (Figures 4A,B). The shape of HA and HA-AI composite was a characteristic slight agglomerated nanosphere shape, and the result was in accordance with the polydispersity index of HA and HA-AI composite. The result showed that incorporation of AI into HA increased the size of the HA-AI composite related to HA and however, does not affect the morphology. The HR-TEM analysis also confirmed that the HA and HA-AI composites were spherical in shape and their size were 106.21 and 177.90 nm, respectively (Figures 4C,D). The elemental composition of HA, AI, and HA-AI composite was determined by SEM-EDX and results were depicted in Figures 4E–G. The HA was found to be constituted of elements Ca (46.91%), P (32.14%), and O (19.52%), and AI was constituted of elements O (11.07%), C (83.64%), Cl (2.41%), and K (1.09%). The HA-AI composite found consists of both elemental constituents of HA and AI, which comprise of Ca (34.70%) P (28.88%), O (14.19%), C (19.31%), and Cl (1.07%). Thus, SEM-EDX analysis concluded that HA was successfully composed of constituents of AI (Table 1).

**Table 1:** Elemental analysis of hydroxyapatite (HA), *A. indica* (AI), and HA-AI composite by SEM-EDX.

Elements	HA	AI	HA-AI composite
Ca (%)	46.91	–	34.70
P (%)	32.14	–	28.88
O (%)	19.52	11.07	14.19
C (%)	–	83.64	19.31
Cl (%)	–	2.41	1.07
K (%)	–	1.09	
Total (%)	98.57	98.21	98.15

### Antioxidant Potential of HA-AI Composite

**Figure 5:**
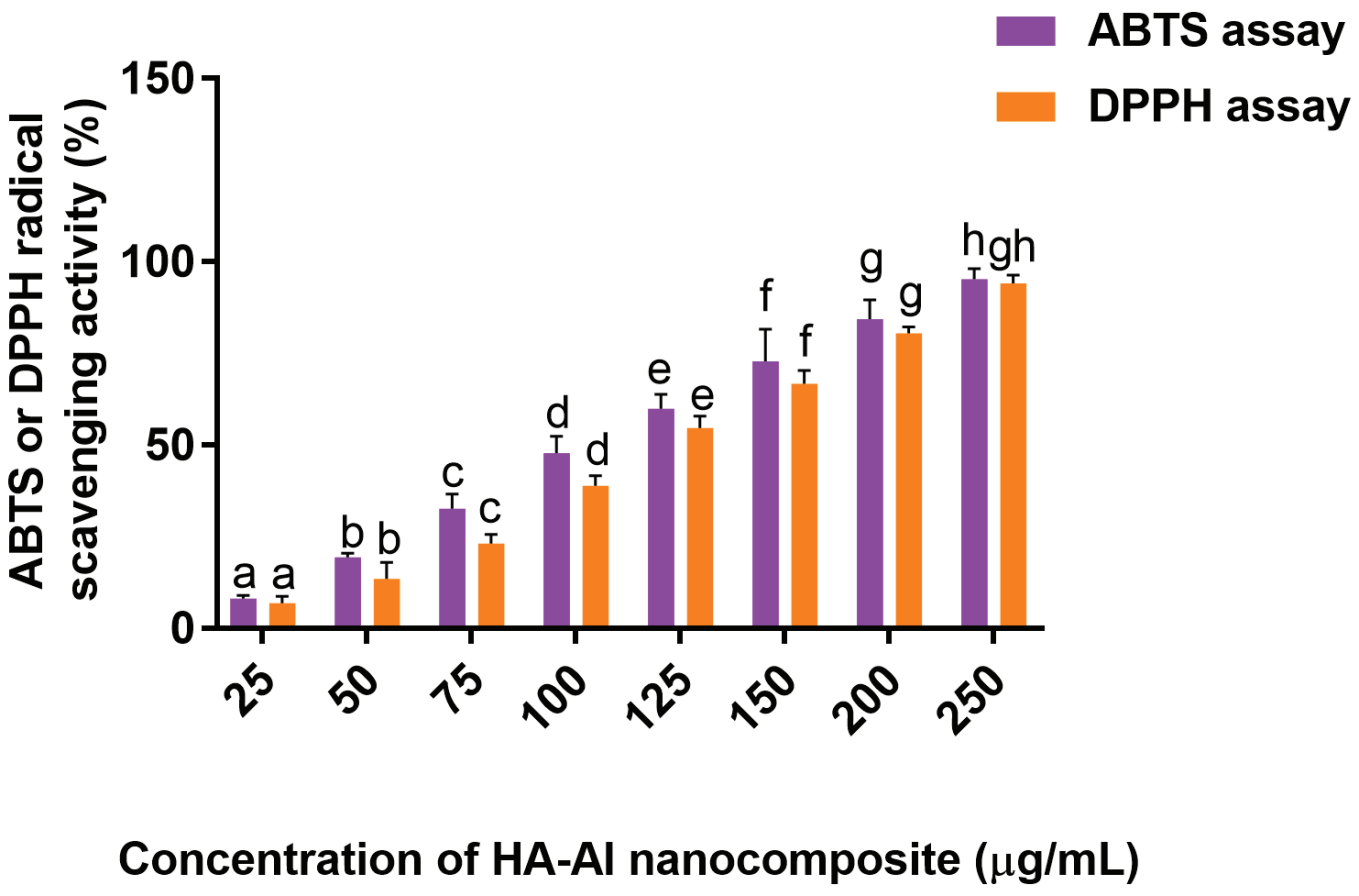
ABTS and DPPH radical scavenging potentials of hydroxyapatite–*A. indica* (HA-AI) composite. The experiments were performed individually in six replicates and achieved results were stated as mean ± standard deviation. The data were statistically evaluated by one-way ANOVA following Tukey’s *post hoc* multiple comparison test. The statistical variance among the experimental groups was measured as significant at *p* < 0.05. The columns with different alphabets were significant within the respective study.

The radical scavenging potential of the HA-AI composite was determined by ABTS and DPPH radical scavenging assays and obtained results were shown in Figure 5. The HA-AI composite exhibited dose-dependent radical scavenging activity and EC50 values (dose required to inhibit about half of radicals) for ABTS and DPPH assays were determined as 115.72 ± 2.33 and 128.51 ± 1.04 μg/ml, respectively. In support of our study, most recently, Sumathra et al. (2018) loaded plant compound 6-gingerol into phosphorylated chitosan armed HA composite and noticed DPPH radical scavenging activity up to 76.16% and reported its biocompatibility for advancing activity on osteoblast and osteosarcoma cells. In our study, a relevant antioxidant activity of the HA-AI composite was noticed, and it could be due to phenolics and flavonoids of AI (Sultana et al., 2007; Choudhary and Swarnkar, 2011). Mostly, environmental pollutants and microbial toxins exhibit toxic effects through the generation of oxidative stress (Zablotsky et al., 1992; Nason et al., 2009). Therefore, the developed HA-AI composite could be highly applicable as an antioxidant in orthopedic and dental implants (Ramakrishnan et al., 2010).

### Microbicidal Activity of HA-AI Composite

The antimicrobial activity of the developed HA-AI composite was tested on selected pathogenic bacteria by micro-well dilution assay as per approved technique of CLSI. The MIC and MBC of HA-AI composite on *S. aureus* (ATCC 700699) and *E. coli* (ATCC 10536) were correspondingly determined as 266.7 ± 28.87 and 600.0 ± 50.0 μg/ml, and 400.0 ± 86.6 and 816.7 ± 76.38 μg/ml. In the interim, MIC and MBC values of standard antibacterial agent tetracycline on *S. aureus* (ATCC 700699) and *E. coli* (ATCC 10536) were determined as 10.32 ± 0.88 and 16.06 ± 1.02 μg/ml, and 22.90 ± 2.40 and 38.32 ± 2.04 μg/ml, respectively. The determined antibacterial activity of HA-AI composite was lower compared to standard tetracycline.

**Figure 6:**
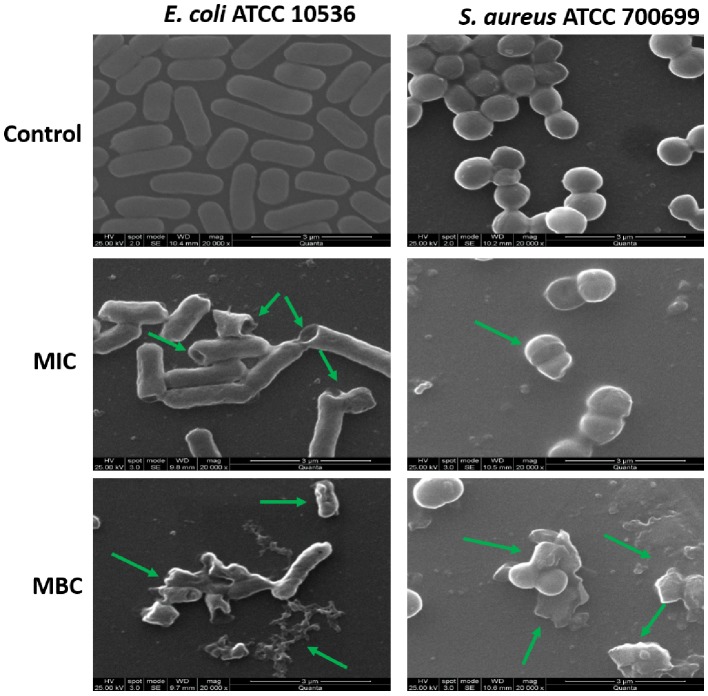
SEM images of *S. aureus* (ATCC 700699) and *E. coli* (ATCC 10536) as untreated (control) and treated with minimum inhibitory concentration (MIC) and minimum bactericidal concentration (MBC) of hydroxyapatite–*A. indica* (HA-AI) composite.

Furthermore, the antimicrobial potential of the HA-AI composite on the micromorphology of bacteria was confirmed by SEM (Figure 6). The HA-AI composite untreated (control) bacteria exhibited characteristic healthy morphology, i.e., smooth and regular surface, whereas bacteria treated with MIC and MBC of HA-AI composite showed detrimental changes in micromorphology, i.e., irregular surface, vesicles, and cellular debris. The evidential detrimental changes in the micromorphology of bacteria were noticed at MBC related to MIC.

In support of our study, Kalaiselvi et al. (2018) synthesized HA nanorods using the flower extract of *Moringa oleifera* and observed the potent antibacterial activity on Gram-positive bacteria (*Bacillus subtilis*, *Monococcus luteus*, and *S. aureus*) and Gram-negative bacteria (*Klebsiella pneumoniae*, *Pseudomonas aeruginosa*, and *Salmonella paratyphi*). Likewise, Scatolini et al. (2018) tested biomimetic HA with different Brazilian propolis and noticed potent antibacterial activity on *S. aureus* ATCC 25923 in the range of >12.5 to >100 μg/ml for MIC and >206.7–800 μg/ml for MBC. Gopi et al. (2013) also synthesized green template assisted HA nanorods using tartaric acid from sources such as banana, grape, and tamarind, and showed a potent antibacterial activity against *E. coli* and *Klebsiella* sp. at a concentration of 100 μl. Similarly, Lakshmeesha et al. (2019) biofabricated zinc oxide nanoparticles with flower bud extract of *Syzygium aromaticum* and demonstrated a potent antifungal and antimycotoxin activity on *F. graminearum*, and Gunti et al. (2019) phytofabricated selenium nanoparticles with fruit extract of *Emblica officinalis* and showed their potent antibacterial activity on foodborne pathogens including bacteria (*E. coli, L. monocytogenes*, *S. aureus*, and *E. faecalis*) and fungi (*A. brasiliensis, A. flavus*, *A. oryzae*, *A. ochraceus*, *F. anthophilum*, and *R. stolonifer*).

The HA is highly biocompatible and will not have antimicrobial property (Zablotsky et al., 1992). In our study, HA-AI composite exhibited potent antimicrobial activity and it could be by phytochemical constituents of AI. Correspondingly, several reports evidently demonstrated the antimicrobial activity of phytoconstituents of AI (Almas, 1999; Thakurta et al., 2007; Joshi et al., 2011). Our study suggests that HA successfully gained antimicrobial activity from AI through biomimetic and, thereby, could find a potential role as an antimicrobial agent in orthopedic and dental implants.

### Therapeutic Potential of HA-AI Composite on LPS-Induced Inflammatory Stress

**Figure 7:**
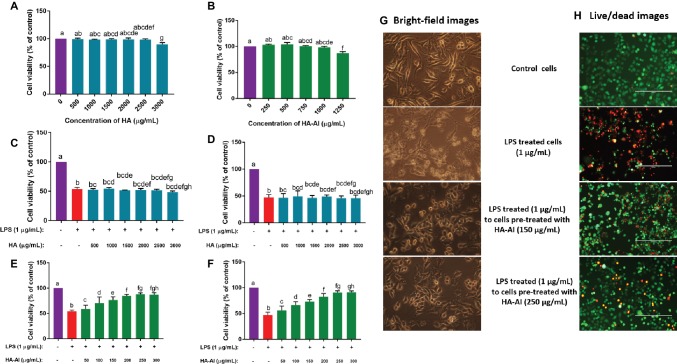
Assessment of protective efficacy of hydroxyapatite (HA) and hydroxyapatite–*A. indica* (HA-AI) composite on LPS-induced death of RAW 264.7 cells by cell viability assays. Biocompatibility of **(A)** HA and **(B)** HA-AI composite on cell viability determined by MTT assay. Protective efficacy of HA against LPS-induced cell death determined by **(C)** MTT and **(D)** live/dead dual staining assays. Protective efficacy of HA-AI composite against LPS-induced cell death determined by **(E)** MTT and **(F)** live/dead dual staining assays. **(G)** Bright-fielding microscopic images representing the protective efficacy of HA-AI composite on LPS-induced cell death. The images were taken at magnification of 400×. **(H)** Live/dead dual staining microscopic images representing the protective efficacy of HA-AI composite on LPS-induced cell death. The scale bar is 200 μm. Green and red colors represent the live and dead cells, respectively. The experiments were performed individually in six replicates and achieved results were stated as mean ± standard deviation. The data were statistically evaluated by one-way ANOVA following Tukey’s *post hoc* multiple comparison test. The statistical variance among the experimental groups was measured as significant at *p* < 0.05. The columns with different alphabets were significant within the respective study.

At first, the effects of different concentrations of HA and HA-AI composite on *via*bility of RAW 264.7 cells were assessed by cell viability assay, i.e., MTT assay. Both HA and HA-AI composite were found to be highly biocompatible, and HA and HA-AI composite correspondingly exhibited a significant cytotoxicity after 2.50 and 1.00 mg/ml related to control (Figures 7A,B).

The LPS-induced inflammatory response was attenuated with pre-treatment of RAW 264.7 cells with different concentrations of HA and HA-AI composite. Thus, ≤3 and 1 mg/ml of HA and HA-AI composite were chosen to assess their therapeutic potential towards the inflammatory response of LPS (1 μg/ml). The cells pre-treated with HA (up to 3 mg/ml) were not found to be cytoprotective against LPS-induced cell death (Figures 7C,D). Whereas, cells pre-treated with the HA-AI composite showed an attenuated response against LPS-induced cell death. The cell viability assays, i.e., MTT and live/dead cell dual staining assays revealed that the HA-AI composite was effective in protecting the cells from LPS-induced inflammatory death, and this characteristic was determined to be dose-dependent (Figures 7E,F). The superlative protective activity of HA-AI composite was noticed at 250 μg/ml. The bright-field microscopic images of cells were depicted in Figure 7G, and images evidently showed that cells treated with LPS (1 μg/ml) produced detrimental morphological changes, i.e., disruption of characteristic cell morphology, disruption of cell membrane, and formation of apoptotic bodies and cellular debris. Whereas cells pre-treated with HA-AI composite survived from the LPS-induced death and optimum protection was noticed at 250 μg/ml of HA-AI composite. The other cell viability assay, live/dead dual staining cell images were depicted in Figure 7H. The cells treated only with LPS (1 μg/ml) evidently showed a greater number of dead cells (red) related to control, whereas cells pre-treated with HA-AI composite survived from LPS-induced death and a greater number of live cells (green) were perceived to be related to LPS-treated cells. In accordance to bright-field microscopic images, optimum protection of HA-AI composite against LPS-induced death was noticed at 250 μg/ml. In conclusion, cell viability assays determined that the HA-AI composite was effective in protecting the cells from LPS-induced death and while HA does not have cytoprotective activity. Thus, we can conclude that AI has successfully imparted protective efficacy to HA *via* biomimetic.

**Figure 8:**
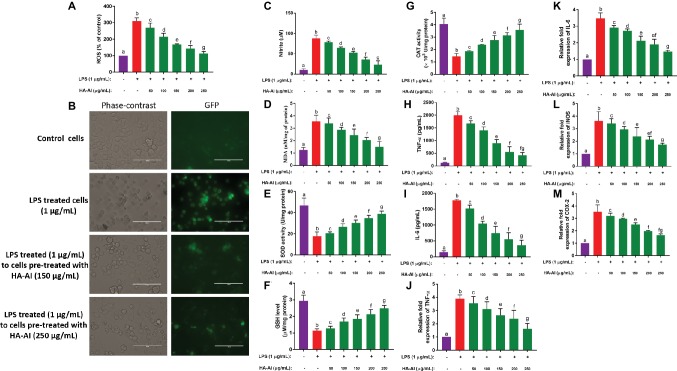
Assessment of protective efficacy of hydroxyapatite–*A. indica* (HA-AI) composite on LPS-induced oxidative and inflammatory stress in RAW 264.7 cells. **(A)** Dose-dependent protective efficacy of HA-AI composite on LPS-induced intracellular ROS generation by DCFH-DA staining. **(B)** Phase contrast and fluorescent microscopic images depicting the protective efficacy of HA-AI composite on LPS-induced intracellular ROS generation by DCFH-DA staining. The scale bar is 100 μm. **(C)** Dose-dependent protective efficacy of HA-AI composite on LPS-induced nitrite generation. **(D)** Dose-dependent protective efficacy of HA-AI composite on LPS-induced lipid peroxidation. **(E–G)** Dose-dependent protective efficacy of HA-AI composite on LPS-induced detrimental effect on antioxidant enzymes, i.e. SOD, GSH, and CAT. **(H,I)** Dose-dependent protective efficacy of HA-AI composite on LPS-induced inflammatory cytokines (TNF-α and IL-6) by ELISA. **(J–M)** Dose-dependent protective efficacy of HA-AI composite on LPS-induced inflammation regulatory genes (TNF-α, IL-6, iNOS, and COX-2) by real-time PCR analysis. The experiments were performed individually in six replicates and achieved results were stated as mean ± standard deviation. The data were statistically evaluated by one-way ANOVA following Tukey’s *post hoc* multiple comparison test. The statistical variance among the experimental groups was measured as significant at *p* < 0.05. The columns with different alphabets were significant within the respective study.

Next, the protective efficacy of the HA-AI composite on oxidative and inflammatory stress induced by LPS is depicted in Figure 8. The HA-AI composite exhibited dose-dependent protective character against intracellular ROS molecules generated by LPS (Figure 8A). The phase-contrast and fluorescent microscopic images of cells exhibiting the protective efficacy of HA-AI composite on LPS-induced oxidative stress is shown in Figure 8B. The images evidently showed higher intensity (fluorescence) of oxidative stress in LPS-treated cells related to control. Whereas cells pre-treated with HA-AI composite showed an attenuated response towards oxidative stress generated by LPS related to cells treated alone with LPS and greater protective efficacy of HA-AI composite against LPS was noticed at 250 μg/ml. Likewise, the HA-AI composite exhibited dose-dependent protective efficacy against nitrite generation and lipid peroxidation induced by LPS (Figures 8C,D). Moreover, HA-AI composite effectively protected the antioxidant enzymes (SOD, GSH, and CAT) from LPS-induced detrimental effects, and it was also found to be dose dependent (Figures 8E–G). Thus, HA-AI composite successfully protected the cells from oxidative stress induced by LPS through safeguarding the antioxidant enzymes. Next, the inflammatory regulatory factors, TNF-α and IL-6, were quantified by ELISA. The results showed that the cells pre-treated with HA-AI composite attenuated generation of TNF-α and IL-6 induced by LPS related to cells treated alone with LPS and was found to be dose-dependent in accordance with the outcomes of oxidative stress (Figures 8H,I). Furthermore, RT-PCR analysis concluded that HA-AI composite successfully down-regulated the expression of inflammatory regulatory genes (TNF-α, IL-6, iNOS, and COX-2) induced by LPS (Figures 8J–M). Thus, oxidative and inflammatory studies concluded that the HA-AI composite protected the cells from LPS-induced inflammatory stress.

**Figure 9:**
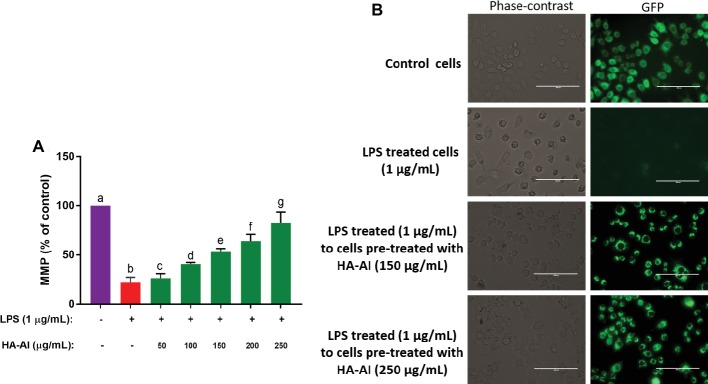
Assessment of restoring efficacy of hydroxyapatite–*A. indica* (HA-AI) composite on LPS-induced depletion of mitochondrial membrane potential (MMP) in RAW 264.7 cells by rhodamine 123 staining. **(A)** Dose-dependent restoring efficacy of HA-AI composite on LPS-induced depletion of MMP. **(B)** Phase contrast and fluorescent microscopic images depicting the restoring efficacy of HA-AI composite on LPS-induced depletion of MMP. The scale bar is 100 μm. The experiments were performed individually in six replicates and achieved results were stated as mean ± standard deviation. The data were statistically evaluated by one-way ANOVA following Tukey’s *post hoc* multiple comparison test. The statistical variance among the experimental groups was measured as significant at *p* < 0.05. The columns with different alphabets were significant within the respective study.

In another study, LPS has significantly depleted the MMP levels of cells related to control and, thus, concluded that LPS might induce cell death through depletion of MMP and obstruction of ATP synthesis. Whereas cells pre-treated with HA-AI composite protected the cells from depletion of MMP induced by LPS, and were also found dose-dependent on results of cell viability, and oxidative and inflammatory assays (Figure 9A). The phase-contrast and fluorescent microscopic images of MMP assay were depicted in Figure 9B. The images evidently showed the restoration of fluorescence (MMP) in cells pre-treated with HA-AI composite related to cells treated alone with LPS. Thus, assays concluded that the HA-AI composite aided in protecting the cells from LPS-induced cell death.

**Figure 10:**
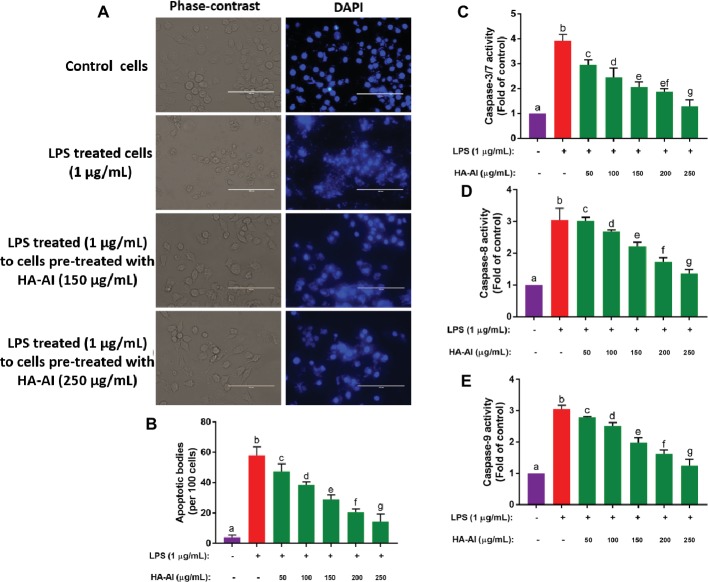
Assessment of protective efficacy of hydroxyapatite–*A. indica* (HA-AI) composite on LPS-induced nuclear damage and apoptosis in RAW 264.7 cells. **(A)** Phase contrast and fluorescent microscopic images depicting the protective efficacy of HA-AI composite on LPS-induced nuclear damage by DAPI staining. **(B)** Dose-dependent protective efficacy of HA-AI composite on LPS-induced apoptosis by DAPI staining. **(C–E)** Dose-dependent restoring efficacy of HA-AI composite on LPS-induced depletion of caspases (3/7, 8, and 9). The experiments were performed individually in six replicates and achieved results were stated as mean ± standard deviation. The data were statistically evaluated by one-way ANOVA following Tukey’s *post hoc* multiple comparison test. The statistical variance among the experimental groups was measured as significant at *p* < 0.05. The columns with different alphabets were significant within the respective study.

In the final study, the protective efficacy of the HA-AI composite on nuclear damage and apoptosis induced by LPS was evaluated by DAPI staining and measurement of caspases 3/7, 8, and 9 (Figure 10). In Figure 10A, LPS detrimentally effaced the nuclear material of cell, resulting in the fragmentation and leakage of nuclear material from nucleus. Whereas cells pre-treated with HA-AI composite protected the cells from nuclear damage induced by LPS. Furthermore, a number of apoptotic bodies were high in LPS-treated cells related to control. On the other hand, fewer apoptotic bodies were noticed in cells pre-treated with HA-AI composite related to cells treated alone with LPS (Figure 10B). Moreover, LPS treated to cells pre-treated with HA-AI composite exhibited significantly low levels of caspases (3/7, 8, and 9) related to cells treated alone with LPS (Figures 10C–E). Thus, overall studies determined that HA-AI composite protected the nuclear material of cells from LPS-induced stress and regulate the apoptotic death through maintenance of caspase levels.

To the best of our knowledge, our study is first report that demonstrated the anti-inflammatory activity of biomimetic HA. However, earlier reports evidently proved the anti-inflammatory activity of various extracts of AI. The researchers reported that phenolics and flavonoids of AI were significantly inhibiting the functions of TNF-α, IL-6, iNOS, COX-2, and NFκB of macrophages and neutrophils relevant to the inflammatory response. The major phenolics and flavonoids responsible for anti-inflammatory responses are nimbin, nimbidin, azadirachtin, quercetin, salannin, gallic acid, catechin, and epicatechin (Atawodi and Atawodi, 2009; Schumacher et al., 2011; Alzohairy, 2016). Moreover, in support of our study, researchers proved that phenolics and flavonoids of other plant extracts were highly efficient in contrasting inflammatory responses (Kalaiselvi et al., 2013; Alvarez-Suarez et al., 2017; Gasparrini et al., 2017). In our study, HA alone did not show protective effectiveness against LPS-induced inflammatory stress. Whereas, the HA-AI composite presented potent anti-inflammatory activity against LPS-induced stress. It can be concluded that the observed anti-inflammatory activity of HA-AI composite could be due to phenolics and flavonoids of AI. In conclusion, our study demonstrated that antioxidant, antibacterial, and anti-inflammatory proficiency could be presented to HA *via* biomimetic with plant sources.

## Conclusion

Ethanolic extract of AI was found rich with phenolics and flavonoids, and determined to be a potential contender for biomimetic of nanomaterials. The HA was synthesized by sol-gel method and HA was biomimetic with AI to form HA-AI composite. The FTIR concluded that the as-synthesized HA-AI composite was successfully comprised of constituents of HA and AI. The XRD pattern and Zeta potential determined that the HA-AI composite was in crystalline phase, has negative charge and could be highly suitable for biological purposes. The SEM showed that the HA-AI composite was spherical in shape, and furthermore, TEM concluded that the HA-AI composite was nano in size. The SEM-EDX concluded that the HA-AI composite comprised elements of HA and AI. The as-synthesized HA-AI composite has presented potential antioxidant, antibacterial, and anti-inflammatory activities against LPS which could be due to phenolics and flavonoids of AI. The HA-AI composite protected the RAW 264.7 cells from LPS-induced cell death through scavenging oxidants, restoring MMP, and upholding inflammatory and apoptotic factors. The study showed that potential beneficial activities were imparted to HA from AI through biomimetic. Thus, the HA-AI composite could be highly applicable as an antibacterial and anti-inflammatory substitute in dentistry and orthopedic.

## Data Availability

The raw data supporting the conclusions of this manuscript will be made available by the authors, without undue reservation, to any qualified researcher.

## Author Contributions

AN and SS have designed the work, executed the experiments, drafted the results, and approved the final version of the manuscript.

### Conflict of Interest Statement

The authors declare that the research was conducted in the absence of any commercial or financial relationships that could be construed as a potential conflict of interest.
