# Supplementary material for: Presentation of Antibacterial and Therapeutic Anti-inflammatory Potentials to Hydroxyapatite via Biomimetic With Azadirachta indica: An in vitro Anti-inflammatory Assessment in Contradiction of LPS-Induced Stress in RAW 264.7 Cells
Source: Front Microbiol. 2019 Aug 7;10:1757. doi: 10.3389/fmicb.2019.01757 (PMC6692563; doi:10.3389/fmicb.2019.01757)
Supplement: Supplementary file 1 [file Table_1.DOCX]

**Supplementary Table 1:** Primer sequences for genes used in RT-PCR

| Gene | Forward sequence (Sense) | Reverse sequence (Antisense) |
| --- | --- | --- |
| TNF-α | GCGACGTGGAACTGGCAGAA | CAGTAGACAGAAGAGCGTGGTG |
| COX-2 | ACCTGGTGAACTACGACTGC | TGGTCGGTTTGATGTTACTG |
| IL-6 | GTTGCCTTCTTGGGACTGAT | CATTTCCACGATTTCCCAGA |
| iNOS | TGGAGCGAGTTGTGGATTGT | CTCTGCCTATCCGTCTCGTC |
| β-actin | TGCTGTCCCTGTATGCCTCTG | GCTGTAGCCACGCTCGGTCA |
